# Pre-siting of UAV stations for traffic accident assessment considering road dispersion

**DOI:** 10.1371/journal.pone.0316431

**Published:** 2025-02-06

**Authors:** Sizhe Wang, Yanying Shang

**Affiliations:** School of Economics and Management, Xi’an University of Technology, Xi’an, China; Beijing Institute of Technology, CHINA

## Abstract

This paper investigates the issue of pre-site selection for drone stations with the aim of enhancing the rapid assessment capability of urban road traffic accidents. Firstly, the influence of traffic accidents on urban traffic is analyzed, and the potential application of drones in rapid response at the accident scene is explored. A minimization model is constructed with the goal of minimizing the cost of accident handling and reducing traffic congestion. To solve this problem, we improved the simulated annealing algorithm by combining the multi-neighborhood strategy, adaptive neighborhood size, and adding a taboo list, and verified the effectiveness of the algorithm. The validity of the model is tested through simulation examples, and the impact of the drone coverage radius and the distribution of accident points on the model performance is explored through sensitivity analysis, providing management insights for the pre-site selection of drone stations.

## 1 Introduction

Global economic growth and rapid urbanization have intensified traffic congestion. In 2023, direct property losses from traffic accidents in China reached 1.24 billion yuan, with 294.27 million private automobiles nationwide, an increase of 15.53 million from the previous year. The surge in private cars has exacerbated issues like "rush hour" congestion. Moreover, studies indicate a significant correlation between traffic congestion and rear-end collision rates [1]. Studies indicate a strong link between traffic congestion and rear-end collisions, underscoring the critical need to address this issue.

Traffic congestion is classified into two types: recurrent and non-recurrent. Recurrent congestion, driven by factors like increased travel demand, limited road capacity, or poor signal coordination, occurs periodically. Non-recurrent congestion, often caused by emergencies like traffic accidents, accounts for roughly one-quarter of total congestion. Delays in assessing and managing accidents can worsen congestion and lead to secondary accidents. Studies indicate that reducing accident assessment time by one minute can cut vehicle delays by about five minutes [2]. Reducing accident assessment time directly decreases vehicle delays, making swift and effective crash assessments crucial for alleviating non-recurrent congestion. Despite the fact that most minor accidents can be quickly resolved, traditional on-site handling methods may still lead to delays. At certain times, even minor delays can cause a lot of unnecessary traffic congestion. The quick response of drones helps shorten the time for accident assessment, thereby reducing the rate of secondary accidents and the degree of traffic congestion. This method provides a new approach to improving traffic congestion caused by traffic accidents by enhancing resource allocation.

In the traditional traffic accident evaluation process, a witness or involved party first notifies traffic authorities via an emergency call. Traffic police or law enforcement then arrive at the scene to implement safety controls, preventing further injuries. Preserving the accident scene and collecting evidence, such as detailed records and photographs of vehicle positions, damages, and road marks, are crucial steps. The police then conduct a preliminary investigation, interviewing witnesses and involved parties and gathering possible video surveillance. In some cases, expert technical analysis and accident reconstruction may be required. Finally, based on the collected evidence, responsibility is assessed, leading to a formal accident report that serves as the basis for legal proceedings and insurance claims. In contrast, using drones in traffic accident assessments can significantly enhance the efficiency and safety of data collection. Upon receiving the accident report, the traffic management center deploys a drone to the scene. Upon arrival, the drone uses onboard High Definition(HD) cameras and sensors to capture comprehensive images and scans of the scene, including vehicle positions, damages, and the surrounding environment. These images and data are transmitted in real-time to the command center, allowing traffic police to remotely assess the scene, evaluate the accident’s severity, and make prompt decisions.

The land acquisition for drone stations might bring about new challenges for deployment, encompassing multiple aspects such as urban planning, coordination among government departments, and allocation of public resources. In practical operations, the methods of land acquisition are diverse, specifically including: making use of existing public infrastructure, for instance, establishing drone stations on government buildings, police stations, or fire stations, etc. These locations usually have superior geographical conditions and complete facilities, which helps to reduce the cost and complexity of establishing new stations; collaborating with private enterprises or properties to set up stations on rooftops or parking lots of shopping malls, companies, etc. This approach enhances the flexibility and coverage of the stations; the government acquires land or leases land. In areas requiring special layouts, the government can obtain suitable land for establishing drone stations through expropriation or leasing. Despite the fact that these areas offer extensive deployable locations, the maintenance challenges resulting from different land natures still require further research and planning.

Strategic siting of UAVs is crucial for ensuring rapid response to future incidents. However, the unpredictable spatial distribution of future traffic incidents presents significant challenges in UAV pre-siting. This study addresses the question of how to optimally deploy a limited number of UAVs in advance to mitigate potential non-recurring congestion and secondary accidents. In the context of rapid traffic incident assessment, we propose a problem focused on early UAV deployment siting aimed at strategically positioning UAVs to effectively reduce non-recurring congestion and secondary accidents.

The necessity of this study arises from the critical importance of response time in current traffic accident handling. Traditional accident handling methods are limited by personnel and physical conditions, making it difficult to cover all accident sites, especially during peak traffic hours or in remote areas. Drone technology, as an efficient supplementary means, can significantly improve accident response speed and reduce traffic congestion and secondary accidents. Our study specifically addresses this practical need by proposing a site selection model based on drones to optimize the coverage range and response time. Our study has two significant innovations: First, the improved algorithm we proposed combines the "multi-neighborhood strategy" and "adaptive neighborhood size" to optimize the deployment requirements of drones and enhance the application effect in complex scenarios. Second, we validated the effectiveness of the model in extreme situations through large-scale simulations, which has not been fully explored in existing literature. Therefore, our study not only provides theoretical support for the application of drones in traffic accident management, but also provides practical guidance for actual deployment.

## 2 Literature review

This paper focuses on the literature most closely related to the rapid assessment of UAV pre-positioning accidents under uncertainty. In Section 1.1, we focus on the literature on the application of UAVs in transportation and logistics. Section 1.2 is devoted to the literature related to UAV facility siting issues. Section 1.3 examines the literature on the uncertain optimization of UAVs. Finally, in Section 1.4, we examine research gaps.

### 2.1 Application of UAV for traffic monitoring

In the research domain of unmanned aerial vehicle (UAV) traffic monitoring applications, scholars have put forward multiple innovative approaches to optimize the performance and safety of UAVs in traffic monitoring. For example, Nagrare et al. [3] studied the traffic management of UAVs in low-altitude airspace and proposed an intersection planning algorithm to solve the conflict problem in the path of UAVs through lane transformation. On the other hand, Lu et al. [4] focused on the application of UAVs in traffic data collection and developed a deep learning-based error compensation scheme to improve the accuracy of road user positioning. Li et al. [5] proposed a framework for extracting high-precision traffic data using UAV videos, which significantly improves the accuracy and efficiency of data extraction through improved object detection and tracking algorithms. On the other hand, Quan et al. [6] investigated the safe radius design of UAVs under communication uncertainty conditions to avoid collisions with obstacles. On the other hand, Mi et al. [7] extracted the kinematic variables of traffic flow from videos captured by UAVs, providing a traffic monitoring method that does not require the vehicle to install any additional equipment. Shan et al. [8] explored a new approach to improve the safety of UAV operations using vehicle-to-vehicle (V2V) communication, demonstrating the possibility of UAV information sharing and autonomous flight coordination.

Within a broader commercial and regulatory framework, Alkadi et al. [9] proposed a decentralized Unmanned Aerial Vehicle (UAV) traffic management system (UTM) that leverages blockchain and smart contract technology to ensure safety and efficiency. Ashqer et al. [10] improved data-driven decision-making for traffic management by evaluating the effectiveness of signalized intersections with data collected by UAVs. Gao et al. [11] investigated the routing problem of collaborative truck and UAV delivery to optimize delivery efficiency in the logistics industry. In addition, Yang et al. [10] combined a joint learning framework and imitation learning techniques to optimize the coordinated actions of a swarm of UAVs through generative adversarial imitation learning (GAIL) and self-imitation learning (SIL) models, demonstrating the advantages of applying this strategy in UAV traffic monitoring scenarios. Reyes-Muñoz et al. [12] explored the air traffic management problem when UAVs are integrated into non-segregated airspace, focusing on the impact on the workload of air traffic controllers when UAVs encounter unexpected events, and verified that the intent-to-fly technique improves situational awareness through simulation experiments. On the other hand, Liu et al. [13] focuses on UAV swarm-assisted edge computing systems, which significantly improve the system performance through data offloading and two-layer optimization strategies. Mahajan et al. [14] used traffic data captured by UAVs to support micro-traffic analysis and optimized data quality through advanced data processing techniques. Zhong et al. [13] improved the route planning and airport siting for UAV inspection by particle swarm optimization algorithm, while Zhou et al. [15] used UAV surveillance data to analyze traffic conflicts, both of which provide new perspectives and tools for urban traffic management.

Overall, these researches not only enhance the application potential of UAV in logistics and traffic monitoring, but also address the crucial challenges regarding safety, efficiency and data accuracy through technological innovation, showcasing the significant role of UAV technology in future traffic monitoring.

### 2.2 Applications of UAV in traffic logistics and their deployment

In the research field of drone deployment and application, various research teams have proposed diversified solution strategies to address specific challenges, covering key areas such as logistics optimization, communication enhancement, environmental monitoring and safety management. By synthesizing these research results, it is possible to observe how UAV technology is gradually addressing the limitations of traditional approaches and opening up new application prospects.

In terms of logistics optimization, Zhou et al. [16] conducted an in-depth study on the application of UAVs in complex logistics systems, which significantly improved the operational efficiency of UAVs and the stability of the system by optimizing the loading strategy and the selection of rescue locations. In addition, Li et al. [17] proposed a set of UAV traffic management frameworks, including path planning and airspace resource allocation in the context of urban low-altitude parcel delivery, which enhances the application of UAVs in urban environments. In the field of communication enhancement. Li et al. [18] utilize UAVs as mobile base stations, which effectively alleviate the capacity pressure of cellular networks by optimizing the deployment location and resource allocation. Fan et al. [19] On the other hand, it optimized the traffic offloading of UAVs through a two-layer network graph model from the perspective of network structure. It improved the overall performance of the network. Finally, in terms of safety management. Beg et al. [20] studied the application of drones in urban infrastructure, demonstrating the potential of drones in emergency response and traffic monitoring, providing new solutions for safety management in smart cities. These studies demonstrate the prospects for a wide range of UAV applications in traffic monitoring, path planning, logistics and distribution, intelligent transportation, and emergency response, providing a variety of optimization algorithms and management frameworks that significantly enhance the efficiency and effectiveness of UAV technology in practical applications.

### 2.3 Optimization considering uncertainty

In the current research literature, a series of innovative solutions and models have been proposed by many scholars for the UAV uncertainty optimization problem, and these researches broadly cover a wide range of fields such as UAV communication, task assignment, path planning, and dynamic environment response. Duan et al. [21] explored a strategy to improve the efficiency of vehicle queue control for UAVs in dynamic communication environments and reduced the number of vehicle queue controls by optimizing the allocation of communication resources to reduce the speed perturbation to ensure smooth and safe traveling. Yang et al. [22] proposed a deep deterministic policy gradient algorithm based on inverted easy speed obstacles for dynamic obstacles, which effectively improves the decision-making speed and accuracy of multi-UAV route planning. Zhang et al. [23] optimized multi-UAV task allocation by using a fuzzy chance-constrained programming model and multi-strategy Gray Wolf optimization algorithm, taking into account the multiple uncertainties of task execution.

In more specific technical applications, Yu et al. [24] propose an improved fireworks algorithm for the uncertainty and multi-objective problems in UAV task assignments, which significantly improves the computational efficiency and accuracy of the algorithm. Xiang et al. [25] focus on the routing and orientation problems of UAVs in uncertain navigation environments and propose an effective memory algorithm to cope with the complex demands of path and orientation optimization. Zhang et al. [26] coped with the wind field interference problem and developed a distributed adaptive control method to ensure the formation stability of UAVs in complex environments. In the multi-user UAV communication system studied by Xu et al. [27], a robust resource allocation algorithm is proposed to optimize power consumption and enhance system performance in response to uncertainties such as wind speed and user position. In addition, the multi-UAV confrontation model proposed by Xu et al. [28] takes into account information uncertainty and provides a new approach to strategy selection for battlefield decision support. Yang et al. [29] An improved velocity obstacle model in dynamic environments effectively enhances the UAV’s obstacle avoidance capability and mission safety. Wang et al. [30] In ISR mission planning, the multi-objective planning problem is solved by transforming the multiobjective planning problem into a single-objective uncertainty programming problem, which addresses the complexity caused by inter-objective dependencies.

In summary, the research on UAV uncertainty optimization covers a wide range of aspects, from communication optimization and task assignment to path planning. The enhancement of each technology is aimed at enhancing the adaptability and effectiveness of UAS in complex environments, which proves the prospect of wide application and practical benefits of modern UAV technology in dealing with uncertainty challenges.

### 2.4 Research gaps

Despite the great potential of UAVs in emergency response, especially in the rapid assessment of traffic accidents, their research in this field is still insufficient compared to their wide application in logistics. Currently, research has mainly focused on the basic functions and technical performance of UAVs and has not yet fully explored their potential for application in traffic accident assessment and handling.

This study aims to construct a model for deploying UAVs in advance for rapid traffic accident assessment and to improve response efficiency and processing accuracy after traffic accidents. The specific application of UAVs in traffic accident scenes has not been widely used, especially in accident reconstruction and assessment. The existing literature lacks a systematic study of the utility of UAVs in collecting data from traffic accident scenes, and how to quickly obtain detailed information about accident scenes through UAVs has not yet been fully explored. There is also a relative lack of research on how to effectively integrate UAVs with the traditional accident handling process and how to optimize this integration process to improve accident response speed and accuracy. Second, although some studies have pointed out the potential of drones in traffic monitoring and management, there is a lack of a clear research framework and mature application examples specifically for real-time data processing and rapid assessment at accident scenes. How to utilize data collected by UAVs for effective accident cause analysis, responsibility determination, and injury assessment are all areas that have not yet been explored in detail in research.

In this study, we hope to effectively improve the response speed of traffic accident handling by constructing a traffic accident-oriented UAV station pre-selection model to provide theoretical and practical support for the future application of UAV technology in the field of traffic safety. The discrete road network is used to represent traffic accidents as demand points in the traffic network, thus facilitating the construction of UAV station deployment. The problem is solved by designing a heuristic algorithm, and the validity of the model is verified. Finally, relevant managerial insights are given to support decision-making based on the analysis of example results and sensitivity analysis of parameters.

## 3 Problem description and model construction

In this chapter, in Section 2.1, we introduced the use of drones in traffic accident assessment. In Section 2.2, we developed an optimization model for selecting a drone station site to minimize the cost of handling traffic accidents and the resulting road congestion, and we also provided a description of the symbols used throughout the paper.

### 3.1 Description of the problem

Drones possess a remarkable temporal advantage in traffic accident processing, particularly in specific circumstances such as areas with high traffic volume or remote regions. Firstly, drones can promptly arrive at the accident site in congested sections or areas with inconvenient transportation, while traditional emergency vehicles (such as motorcycles and cars) are typically constrained by traffic conditions, resulting in delays. Secondly, drones are capable of capturing the entire scene of the accident from multiple aerial perspectives in real time, providing comprehensive and precise data for accident assessment, which is more efficient than ground vehicles. Additionally, the speed and flexibility of drones enable them to cover a broader area and respond to multiple accident points within a short period of time.

The handling of traffic accidents is typically categorized based on the severity of the incident and its consequences, with UAV involvement varying accordingly. Drones can quickly arrive at accident scenes, capture aerial images for a panoramic view, and assess the impact on surrounding traffic to aid in devising diversion plans. In more severe accidents, the high-definition images and videos captured by drones assist in accident analysis and evidence collection, facilitating insurance claims. Additionally, in major incidents, drones support rescue teams by identifying the location, number, and mobility of trapped individuals, assessing rescue difficulty, and evaluating safety risks. These drone applications enhance the efficiency of traffic accident management, reduce road recovery time, and mitigate the societal impact. Table 1 illustrates the classification of traffic accidents by severity and the corresponding degree of UAV involvement in accident assessment.

**Table 1:** Classification of traffic accidents and drone involvement level.

Traffic Accident Rating	Property Damage	Injuries to Personnel	Death of a Person	Chain of Accidents	Level of drone involvement			
					Record of Site Assessment	Traffic Control and Diversion	Analysis of Data Collection	Emergency Relief Operations
Minor Accident	✓				✓			
Average Accident	✓	✓			✓	✓		
Major Accident	✓	✓	✓		✓	✓	✓	✓
Accident of Epic Proportions	✓	✓	✓	✓	✓	✓	✓	✓

The level of drone involvement in traffic accidents varies significantly with the severity of the incident, as does the response time. In practice, the frequency of traffic accidents differs greatly by severity. In 2023, China recorded approximately 1.75 million road traffic accidents, with minor and general accidents constituting around 80% and 15% of the total, respectively. In contrast, larger accidents accounted for just 0.025% and very large accidents for about 0.005% of the total. Given these statistics, the focus when addressing rapid traffic accident assessment is on two primary scenarios: on-site assessment records and traffic monitoring and diversion. For minor accidents, swift resolution can be achieved through self-negotiation or drone-assisted rapid assessment. For general accidents, drones assist traffic police in conducting on-site investigations and quickly assessing the situation to minimize road blockage. Consequently, this paper’s exploration of rapid drone-assisted traffic accident assessment focuses on minor and general accidents.

### 3.2 Modeling

**Fig 1:**
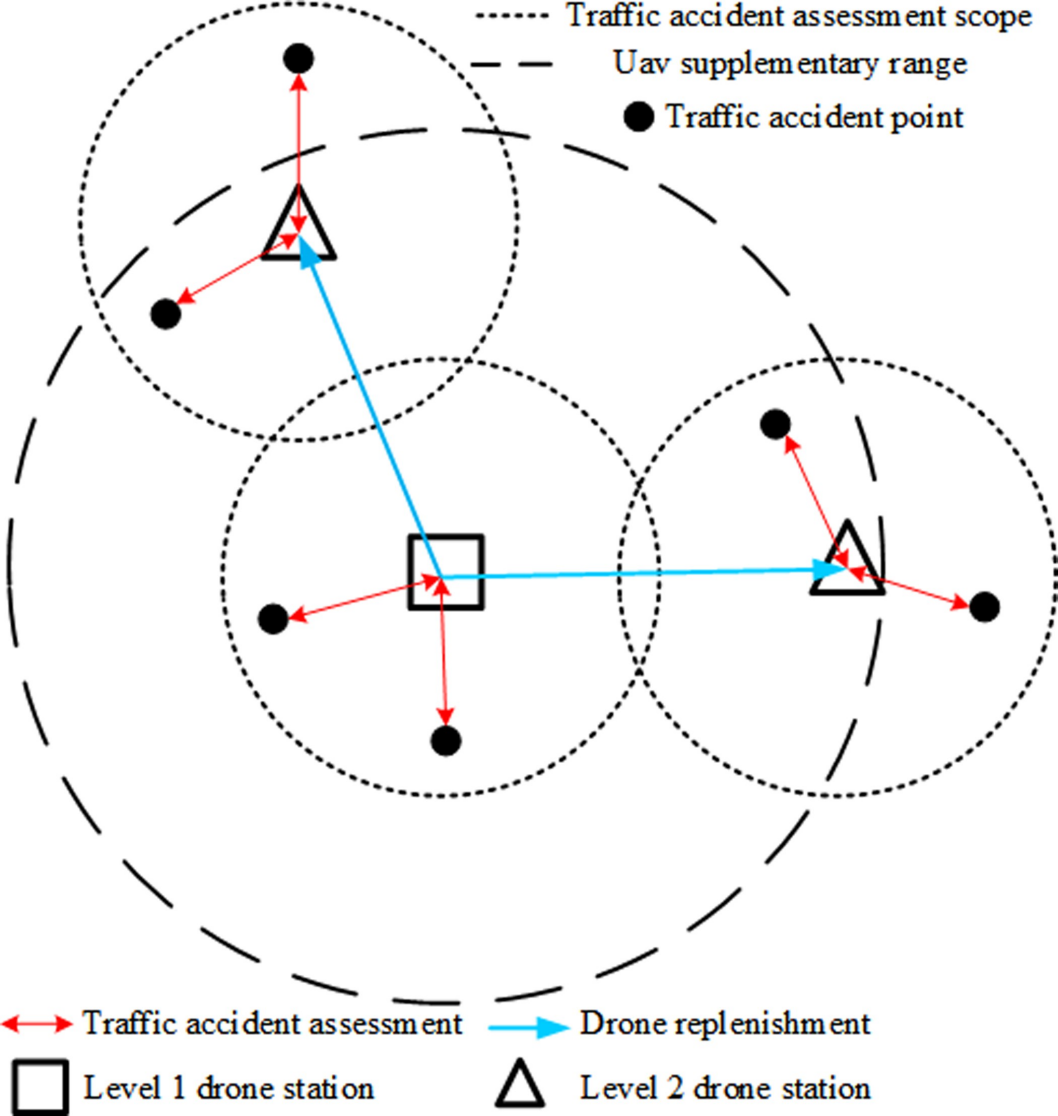
Schematic diagram of a drone station for rapid assessment of traffic accidents.

Traffic accidents are influenced by geographical, climatic, seasonal, temporal, and socio-cultural factors, leading to variations in accident frequency and severity across different regions. To effectively deploy UAVs and UAV stations, it is crucial to consider the local traffic conditions to ensure that the UAV service level meets the demand for accident response. Traffic accident points are located on urban road segments every 2 km, including information on location, time, and classification. Each accident point has a time window; exceeding this window increases traffic congestion and incurs a penalty cost. Given the unpredictability of accidents and the potential for drone failures, UAV stations are categorized into two levels: Level 1 stations, which maintain a sufficient number of drones to support Level 2 stations and conduct accident evaluations, and Level 2 stations, which are solely responsible for accident evaluations. Due to drone endurance limitations, the assessment coverage is restricted to the maximum round-trip distance the drones can travel. Level 1 stations provide supplementary coverage, potentially extending their area of service to twice that of Level 2 stations, as illustrated in Fig 1. Table 2 details the relevant variables.

**Table 2:** Description of variables.

Symbols	Explanation
*P*	A collection of potential Tier 2 site locations
*A*	Accident Location Gathering
*K*	Maximum number of level 2 sites
*R*	A collection of potential Tier 2 site locations
*R*_1_	Supplementary coverage radius for level 1 sites
*C*_*i*_	Capacity of level 2 site *p*_*i*_
*B*	Total budgetary constraints
*c*_*i*_	Construction costs for Level 2 Site *p*_*i*_
*w*_*i*_	Priority of accident point *a*_*i*_
*W*_*i*_	Time window at accident point *a*_*i*_
*P*_*i*_	Probability of accident at accident point *a*_*i*_ (1 for minor accidents and 2 for general accidents)
*L*_*i*_	Accident level at the accident site *a*_*i*_
*T*_*i*_	Response time at the point of incident *a*_*i*_
γ	Penalty cost factor
*d*_*ij*_	Distance from site *p*_*i*_ to accident location *a*_*i*_
*x*_*i*_	Whether a level 2 site*p*_*i*_ is selected. *x*_*i*_∈{0,1}
*x*_*ij*_	Does level 2 site *p*_*i*_ serve the incident site *a*_*i*_ .*x*_*ij*_∈{0,1}
*z*_*i*_	Level 2 station *p*_*i*_ has a traffic accident within 10 minutes. *z*_*i*_∈{0,1}
*s*_*i*_	Whether the level 1 site is selected. *s*_*i*_∈{0,1}
*y*_*i*_	The number of drones assigned to the site *p*_*i*_
*η*	Endurance levels of drones

Traffic accidents are categorized into two levels, minor and general, and the gains and losses from traffic congestion are taken into account. Based on statistical analysis of actual traffic accident data, when accidents do not occur simultaneously within 10 minutes, it is shown that one drone per site is sufficient to cope with the demand. These drone sites play a key role in the rapid assessment and management of traffic accidents, and their level of service is at the center of the issue of pre-determined sites. A key metric for assessing service levels is response time, which is directly related to the efficiency of incident response. One of the objectives of this study is to minimize the expected response time between the UAV station and the accident site to ensure that the UAV can reach the accident site quickly. Considering the constant flight speed of the UAV, we refine this objective to minimize the response distance. Based on the above considerations, the model can be represented as follows:

$$\overset{m}{\underset{j=1}{min\sum_{j=1}^{m}P_{j}\cdot (\overset{n}{\underset{i=1}{min}}(d_{ij}\cdot x_{i}))+\sum_{j=1}^{m}(\alpha \cdot (L_{j}==1)+\beta \cdot (L_{j}==2))\cdot P_{j}+\sum }}\lambda \cdot max\left(0,\frac{d_{ij}}{v}+(L_{j}==1)\cdot C_{1}+(L_{j}==2)\cdot C_{2}-T_{j}\right)$$ (1)

$$\sum_{i=1}^{n}x_{i}\leq K$$ (2)

$$\sum_{i=1}^{n}x_{ij}\geq 1$$ (3)

$$\sum_{j=1}^{m}x_{ij}\leq C_{i}$$ (4)

$$\sum_{i=1}^{n}(cost_{i}\cdot x_{i})\leq B$$ (5)

$$\sum_{i=1}^{n}(w_{j}\cdot x_{ij})\geq W_{j}$$ (6)

$$y_{i}\leq z_{i}+1$$ (7)

$$\sum_{j=1}^{m}x_{ij}\leq E_{i}$$ (8)

The objective function (1) represents the selection of a number of UAV sites in the urban road network such that the weighted average response distance for the UAV to reach the accident scene from these sites is minimized, taking into account the congestion cost due to different levels of traffic accidents and the penalty cost due to the time window constraint. Eq (2) denotes the limit on the number of level 2 sites. Eq (3) denotes the service limit for each accident site, i.e., each site should serve the accident site. Eq (4) denotes the site capacity limitation, i.e., the number of incident points that can be served by each site over time is limited by the UAV range and recharge rate. Eq (5) indicates that the cost of site construction is limited by the budget. Eq (6) indicates that high-priority incidents are prioritized to be served. Eq (7) indicates that only one drone is assigned to each level 2 site if there are no accidents for 10 minutes. Eq (8) indicates that the number of accidents served by drones at each site *p*_*i*_ cannot exceed its range level. In addition, before site selection based on potential drone sites, the location of the potential sites first needs to be determined. With the goal of maximizing the coverage of potential sites, a greedy algorithm is used to select the locations of potential sites so that they cover as many accident points as possible.

**Fig 2:**
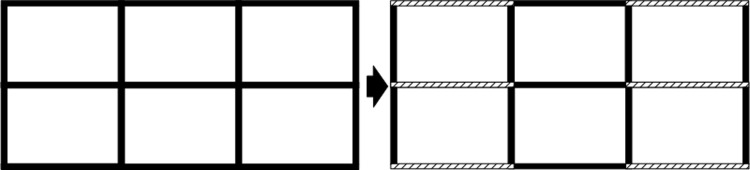
Discretization of road network.

Since traffic accidents occur only on roads and certain road segments have higher accident rates, it is useful to approximate successive accident locations into multiple sub-segments. Based on statistical data, it is possible to divide some of the longer roads into discrete segments, as shown in Fig 2, and by dividing the road network into smaller segments that can be individually treated and numbered, specific accident-prone road segments can be identified and analyzed more efficiently. This approach allows us to focus on areas with high accident rates, enabling targeted interventions and upgraded traffic safety measures.

In fact, despite the existence of obvious high-frequency areas and high-frequency time periods for traffic accidents, in actuality, these areas may have been specially deployed by the traffic management section with additional traffic management personnel for directing and channeling, an act that can significantly reduce the probability of traffic accidents and the speed of handling, but still need to deal with episodic traffic accidents quickly on other roads. Therefore, more UAVs may need to be deployed at level 2 UAV stations at specific times and areas in order to reduce the compliance and pressure on the traffic management system.

## 4 Algorithm design

To address the constructed model, this chapter develops a heuristic algorithm for its resolution. Initially, in Section 3.1, we analyze the model’s complexity and establish that it is an NP-hard problem. In Section 3.2, we outline the algorithmic flow. Furthermore, in Section 3.3, we integrate various algorithm enhancement strategies to optimize solution performance, including: multi-neighborhood strategy, adaptive adjustment of neighborhood range size, and the incorporation of a taboo list.

### 4.1 Complexity analysis

To determine the complexity of the model problem, it is necessary to analyze the model structure and the difficulty of solving it. The model built in this paper belongs to the facility location problem, which is influenced by multiple factors such as response time, cost, traffic accident time window, and drone endurance. The facility location problem is a typical NP-hard problem. Because the facility location problem requires combinatorial optimization, that is, seeking the optimal solution among many possible site combinations. And the facility location problem will have an exponential growth in the number of combinations as the scale increases. Classical solutions include integer linear programming (ILP) or mixed integer linear programming (MILP). In solving the facility location problem to find the worst-case scenario, it is necessary to check all possible facility combinations, which leads to an exponential complexity. In addition, the time window constraint and punishment cost introduced further increased the complexity of the problem. The time window constraint requires that the task be completed within a specific time or a penalty cost will be incurred, adding additional constraints. The drone endurance constraint requires that the drone serve multiple accident sites within a certain time, increasing the number of model variables and constraints, further increasing the difficulty of solving.

### 4.2 Algorithm flow

To solve the drone site selection model, we designed an improved heuristic algorithm for quick response to traffic accidents. As shown in Table 2, the algorithm flow first involves initializing parameters, which includes setting potential sites, accident points, coverage radius, and other key parameters. Then, the algorithm performs multiple random initial site selection to increase the probability of finding the optimal solution by generating multiple initial solutions. In the initial site selection stage, the algorithm uses a greedy algorithm to select the site with the strongest coverage ability until the number of sites or budget limit is reached, thus ensuring the quality of the initial solution. Based on this, the initial assignment stage assigns accident points to the nearest sites to ensure that each accident point is effectively responded to.

In addition, the algorithm optimizes the site selection and accident point allocation through a local search strategy, combining simulated annealing algorithm and multi-neighborhood strategy. During this process, the algorithm adjusts the neighborhood size and uses tabu search technology to avoid circular search. To explore better solutions, the neighborhood solutions are generated by replacing or exchanging sites. For each neighborhood solution, the target function value is evaluated, and whether to accept the solution is judged based on simulated annealing conditions, thereby updating the current solution and target function value. The algorithm controls the search process by gradually lowering the temperature and judges whether to continue searching based on the improvement of the solution and the temperature. Finally, the algorithm calculates the target function value of the optimal solution, including total cost, congestion benefit, and time window penalty, to decide whether to update the optimal solution. After completing the search for all initial solutions, the algorithm outputs the optimal solution and ends the entire process. The algorithm effectively solves the drone site selection problem and demonstrates the efficiency and practicality of optimization algorithms in real-world applications.

### 4.3 Algorithmic improvements

To optimize the selection of drone sites and improve the performance and quality of the algorithm, we made several key improvements to the original algorithm. These improvements include multi-neighborhood strategy [31], adaptive neighborhood size [32], and tabu search [33]. We focused mainly on combining and improving these strategies for the problem of drone site planning, especially for the multi-objective optimization problem of drone site selection. To meet the specific needs of drone site selection, we designed and adjusted these algorithms to better address the uncertain factors such as drone coverage range, response time, and accident distribution. The combination of multi-neighborhood strategy and adaptive neighborhood size enhances the global search ability and local optimization ability of the algorithm, effectively avoiding the problem of local optimal solutions. The tabu search mechanism improves the efficiency of the algorithm by preventing repeated searches. Although these methods have been applied in other fields, the specific combination and optimization application in the field of unmanned airport site planning are one of the main innovations of this paper.

#### (1) Multiple Neighborhood Strategies (MNS)

**Fig 3:**
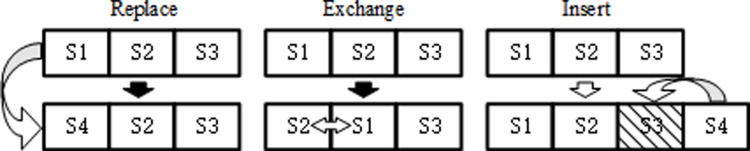
Multi-neighborhood search strategy.

In the problem of drone site selection, the multi-neighborhood strategy improves the quality of the solution and the overall performance of the algorithm by generating diverse combinations of sites through a variety of site combination generation methods, avoiding getting trapped in a local optimum in a single combination strategy. The role of the strategy in the model solving process is to increase the diversity of the search space through multiple different neighborhood generation strategies, thereby enhancing the algorithm’s exploration ability and solution quality. After implementing the site replacement, site exchange, and site insertion strategies, the algorithm significantly enhances the diversity of the search space and effectively prevents the local optimum dilemma in the search process. The use of these strategies not only improves the algorithm’s ability to explore unknown areas, but also significantly improves the overall quality of the solution. As shown in Fig 3, in the specific implementation, a random neighborhood generation strategy is selected for each iteration to generate a new neighborhood solution, ensuring the broadness and effectiveness of the algorithm’s search.

In the algorithmic process, the set of sites is adjusted through three strategies aimed at optimizing the quality of the solution and enhancing the breadth of the search. The specific strategies are as follows: the first one is site replacement, which removes a site from the selected site set and introduces a new potential site, e.g., replacing s1 with s4 in the site set [s1,s2,s3] and updating the set to [s4,s2,s3]. The second strategy is site swapping, which swaps the positions of two sites within the current site set, e.g., swapping the positions of s1 and s2 in [s1,s2,s3] to obtain the new set [s2,s1,s3]. The last strategy is site insertion, an operation in which a site is removed from a set while a new site is added, e.g. replacing s3 with s4 in [s1,s2,s3] results in [s1,s2,s4]. Together, these strategies aim to seek better site configurations and improve the overall performance of the algorithm.

#### (2) Adaptive neighborhood size (adaptive neighborhood size)

In the problem of drone site selection, adaptive neighborhood size can ensure that the algorithm quickly locates a superior site combination in the early stage and further optimizes it through fine-tuning search in the later stage, thus improving the quality of the solution. This strategy improves the search efficiency and solution quality in the model solving process by dynamically adjusting the search range to provide appropriate search strategies for the algorithm at different stages. Specifically, the strategy uses a larger neighborhood range for extensive search in the early stage to explore multiple regions of the solution space. As the iteration progresses, the neighborhood range is gradually reduced to focus on fine-tuning search in promising regions. The adjustment of the neighborhood is based on the change in the performance of the current solution: if the solution is improved during the iteration, the search neighborhood is expanded to enhance exploration ability; if the solution does not show improvement, the neighborhood is narrowed to focus on optimizing the current solution. This strategy effectively balances the relationship between global search and local search, improving the efficiency and overall quality of the solution.

**Fig 4:**
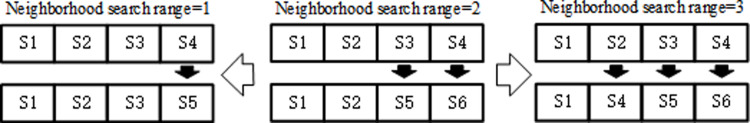
Adaptive neighborhood changes.

During the algorithm optimization, the neighborhood size is adjusted according to the changes in the performance of the current solution to enhance the algorithm’s flexibility and effectiveness. Initially, the neighborhood size is set to 2, meaning that each change involves two sites. During the iteration process, if the current solution is found to be improved compared to the previous one, the neighborhood size will be increased to further expand the search scope to explore more possible solutions, as shown in Fig 4, where the range of the neighborhood change is adjusted from 2 to 3. On the contrary, if the current solution does not show any improvement, the neighborhood size will be reduced to optimize the existing solutions in a more focused way, e.g., from 2 to 1. This dynamic adjustment mechanism helps to find a balance between global search and local refinement, improving the overall performance of the algorithm.

#### (3) Tabu search (taboo search)

In the process of algorithm optimization, the taboo search technique is introduced to prevent the search process from getting stuck in a loop or being limited to only locally optimal solutions, thus enhancing the global exploration capability of the algorithm. By maintaining a taboo list of recently visited solutions or executed taboo operations, the algorithm can effectively avoid repeated exploration of known regions. In a specific implementation, if a candidate’s new solution appears in the taboo list, the solution will be excluded and not accepted. The taboo list is provided with a fixed capacity limit, and once this limit is reached, the earliest recorded taboo operation is removed from the list to make room for a new operation. This strategy ensures a diversity of solutions while facilitating a deeper search for potentially optimal solutions.

**Fig 5:**
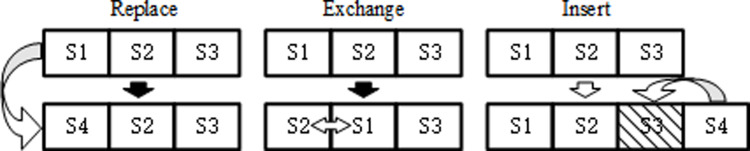
Changes in the prohibited list.

In the taboo search step of the algorithm, as shown in Fig 5, a new solution is first generated and checked if it already exists in the current taboo list. If the new solution is not recorded in the taboo list, it is accepted and added to the taboo list to prevent repeated searches in the future. Subsequently, to maintain efficient management of the taboo list and to ensure that its size does not exceed a preset limit, once the list reaches or exceeds its maximum capacity, the earliest entry is removed from the list to make room for a new solution. This process helps to enhance the exploration efficiency during the search process, avoiding falling into local optima while ensuring that the algorithm continues to progress towards the global optimum.

## 5 Algorithm authentication

This section begins with an overview of the data used in the case study and a detailed discussion of the parameters and details of the calculus in Section 4.1. The study uses Python 3.7 for model solving and runs on a Windows 10 operating system with an Intel(R) Core(TM) i5-11357H@2.40 GHz processor and 16GB of RAM. Section 4.2 evaluates and compares the performance of this algorithm with the conventional algorithm. Section 4.3 explores the impact of various parameters through sensitivity experiments, from which some managerial insights are gained. Overall, this section provides a comprehensive evaluation of our developed UAV pre-positioning model and validates its effectiveness in traffic network incident response.

### 5.1 algorithm description

In order to fully verify the validity of the model at different scales and accident intensities. The parameter settings for the different scale calculations are shown in Table 3 below. The width of discrete roads can be expressed by setting multiples of the horizontal and vertical coordinates of random accident points. Randomized examples are generated considering different potential sites, accident points, coverage, and accident levels, as shown in Table 4, with a view to fully exploring the validity of the model and management insights.

**Table 3:** Algorithm flowchart.

Heuristic algorithm
Input: Potential site locations, accident locations and initial parameters
Output: Relationship between site locations and accident points assigned to sites
1: Select the number of cycles to generate multiple random initial solutions
2: Employ a greedy algorithm to select the initial solution until the site quantity limit or budget limit is reached
3: Conduct local search and optimize the site selection and accident allocation by using simulated annealing and multi-neighborhood strategies;
4: Generate neighborhood solution schemes and optimize the neighborhood solutions through replacement, exchange and insertion strategies.
5: Calculate the neighborhood objective value and update the objective value of the solution scheme
6: Judge whether to accept the neighborhood solution
7: Update the tabu list. If the new solution is accepted, update the tabu list to avoid returning to the recently visited solutions
8: Lower the temperature by multiplying it by the cooling rate and determine whether the stopping condition is satisfied
9: Determine whether all initial solutions have been attempted
10: Output the optimal solution and end the process

**Table 4:** Example parameter configuration table.

Parametric	Small-scale examples	Medium-sized example	Large-scale arithmetic example
Map size	100*100	100*100	100*100
Discrete road widths	2	2	2
Number of accident points	10	20	40
Coverage	2	2	2
accident level	1 and 2	1 and 2	1 and 2

### 5.2 Algorithm performance analysis

In order to solve large-scale arithmetic cases and gradually increase the number of accident points from 40 to 200, 10 at a time, to test the efficiency of the solution under different cross-accident pressures. We will use the Improved Heuristic Algorithm (IHA), the traditional Genetic Algorithm (GA), and the Particle Swarm Algorithm (PSO). The primary initial parameters of each algorithm are presented in Table 5 as follows.

**Table 5:** Comparison of algorithm parameters.

IHA	GA	PSO
Initial neighborhood size: 3	Population size: 100	Particle count: 50
Maximum iteration count: 500	Crossover probability: 0.8	Maximum iteration count: 500
Adaptive neighborhood adjustment frequency: 10 times	Mutation probability: 0.05	Inertia weight (w): 0.9
Taboo list size: 50	Maximum generation: 500	(Linearly decreased to 0.4)
Temperature parameter: initial value 100, cooling coefficient 0.95	Selection strategy: Roulette wheel selection	Individual learning factor (c1): 2

We will solve each algorithm and compare their computation time, optimal solution quality, and number of accident points. The solution results are shown in Table 6 below.

**Table 6:** Comparison of the quality of algorithms to solve the optimal solution.

Number of accident points	IHA	GA	PSO
40	120.5	130.2	128.7
50	135.4	146.3	143.2
60	150.1	161.5	158.1
70	165	176.8	173
80	179.8	192.1	188.3
90	194.7	207.4	203.6
100	209.5	222.7	218.9
110	224.4	238	234.2
120	239.2	253.3	249.5
130	254.1	268.6	264.8
140	268.9	283.9	280.1
150	283.8	299.2	295.4
160	298.6	314.5	310.7
170	313.5	329.8	326
180	328.3	345.1	341.3
190	343.2	360.4	356.6
200	358	375.7	371.9

As the number of accident points increases, the optimal solution quality values of all three algorithms (IHA, GA, PSO) gradually increase, as shown in Table 7. This increasing trend is linear, with a more consistent increase in the solution quality values for all algorithms for each additional 10 accident points. IHA has the lowest solution quality value in all cases, implying that the IHA algorithm performs optimally in solving this problem.GA has the highest solution quality value in all cases, suggesting that GA performs the worst on this problem. PSO has solution quality values between IHA and GA, indicating that PSO performs better than GA but not as well as IHA. The gap between IHA and the other two algorithms (especially GA) is gradually widening as the number of accident points increases, indicating that IHA is more effective in dealing with large-scale problems. When the number of accident points reaches 200, the solution quality value of IHA is 358.0, while that of GA is 375.7 and PSO is 371.9, and the solution quality value of IHA is significantly better than the other two algorithms.

**Table 7:** Comparison of algorithmic solution times(S).

Number of accident points	IHA	GA	PSO
40	5.2	30.4	25.7
50	6.1	34.8	29.3
60	7	39.2	33.2
70	7.8	43.7	36.8
80	8.7	48.1	40.4
90	9.5	52.5	44.1
100	10.3	57	47.8
110	11.2	61.4	51.4
120	12	65.8	55
130	12.9	70.3	58.7
140	13.7	74.7	62.3
150	14.6	79.1	66
160	15.4	83.6	69.6
170	16.3	88	73.3
180	17.1	92.4	76.9
190	18	96.9	80.6
200	18.8	101.3	84.2

The solution times for all three algorithms (IHA, GA, PSO) rise gradually as the number of accident points increases. This upward trend is roughly linear, with a more consistent increase in the solution time for all algorithms for each additional 10 accident points. In all cases, IHA has the lowest solution time, implying that the IHA algorithm is the most efficient at solving this problem.GA has the highest solution time in all cases, suggesting that GA is the least efficient at this problem. PSO has a solution time between IHA and GA, suggesting that PSO is better than GA but not as efficient as IHA. As the number of accident points increases, IHA increases with each other and with each other algorithm (especially GA). In two algorithms (especially GA), the gap between IHA and the other two algorithms is gradually widening, which indicates that IHA is more efficient in dealing with large-scale problems. When the number of incident points reaches 200, the solution time of IHA is 18.8, while that of GA is 101.3, and that of PSO is 84.2, which means that the solution time of IHA is significantly better than the other two algorithms.

In actual circumstances, the deployment of unmanned aerial vehicles (UAVs) can be regarded as a supplementary approach for the existing traffic accident handling systems. Its core capability lies in enabling the gradual enhancement of traffic accident handling capacity through resource investment. Hence, although UAV stations can be pre-planned, in application, once the number of traffic accidents surges abruptly or some extreme situations occur, rapid response planning for UAV deployment is necessary. Consequently, the solution speed and the number of solutions become especially significant in these cases. By comparing the solution speed and the number of solutions, the aim is to verify the stability and efficiency of the model under high-pressure conditions, thereby ensuring that the handling quality and response speed can be enhanced concurrently with the increase in resource investment.

### 5.3 Analysis of results

The results of solving for each scale example are shown in Table 8, where the number of uncovered traffic accident sites and the cost of service are gradually reduced by gradually increasing the number of UAV sites.

**Table 8:** Summary of solutions for each scale example.

Small-scale examples			Medium-sized example			Large-scale arithmetic example		
Maximum number of drone sites	objective function	Number of accident points that cannot be covered	Maximum number of drone sites	objective function	Number of accident points that cannot be covered	Maximum number of drone sites	objective function	Number of accident points that cannot be covered
1	1200	7	1	3600	16	1	3600	30
2	800	6	2	2400	14	2	2400	25
3	600	5	3	1800	13	3	1800	20
4	500	4	4	1200	12	4	1500	16
5	400	3	5	900	11	5	1200	13
6	300	2	6	700	10	6	1000	10
7	200	1	7	600	9	7	900	8
8	100	0	8	500	8	8	800	7
9	100	0	9	450	7	9	700	6
10	100	0	10	400	6	10	600	5
11	100	0	11	350	5	11	500	4
12	100	0	12	300	4	12	400	3
13	100	0	13	250	3	13	300	2
14	100	0	14	200	2	14	200	1
15	100	0	15	150	1	15	100	0
16	100	0	16	100	1	16	100	0
17	100	0	17	50	0	17	100	0
18	100	0	18	50	0	18	100	0
19	100	0	19	50	0	19	100	0
20	100	0	20	50	0	20	100	0

**Fig 6:**
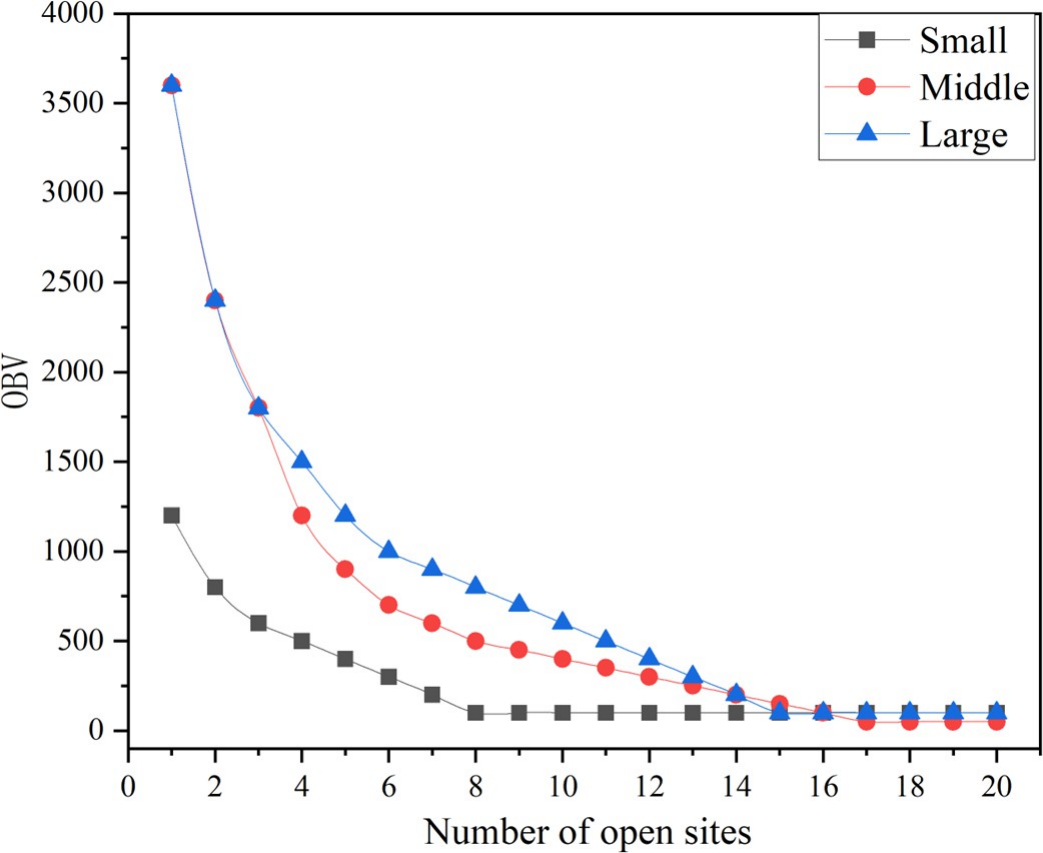
Plot of changes in the objective function for progressive site opening at various scales.

For all three scale scenarios, there is a general trend of decreasing OBV values as the number of open sites increases. There is a sharp initial decline, with OBV values for all scales dropping sharply in the first few open sites. After reaching a certain number of open sites, the OBV values gradually leveled off, with the addition of more sites having a diminishing effect on the OBV values. Comparing the three sizes, the small size has the fastest decline in OBV values and levels off at about 10 open sites. Medium-scale OBV values started higher and declined slightly slower but had a similar overall trend and eventually leveled off as well. Large-scale OBV values started the highest and declined the fastest initially but were still higher than small- and medium-scale at 20 open sites. In addition, during the initial phase (0–5 open sites), all sizes had the fastest rate of decline in OBV values. After the initial phase, the rate of decline slows down, suggesting that adding more sites has diminishing returns in terms of reduced OBV values. As shown in Fig 6, increasing the number of open sites significantly reduces initial OBV values, but this gain diminishes as the number of sites increases further. The large scale has the highest starting OBV value and the most significant initial decrease, but even after 20 sites are open, it still has a higher OBV value than the other two scales.

For all three scales, there was a general downward trend in the number of uncovered sites as the number of open sites increased. For the first few open sites, the number of uncovered sites drops sharply for all scales. After reaching a certain number of open sites, the number of uncovered sites leveled off, and the addition of more sites had a diminishing effect on the number of uncovered sites. Comparing the three sizes, the small size has the fastest decline in the number of uncovered sites and levels off at about 10 open sites, eventually converging to 0. The medium size has a higher starting number of uncovered sites and a slightly slower decline, but the overall trend is similar, also converging to 0. The large size has the highest starting number of uncovered sites and the fastest initial decline, which also converges to 0. The management insights gained from this are that the utility of UAV site deployment is not compromised within a certain interval when the scope and number of incident site requirements increase. Therefore, in the actual deployment process, drone sites should be deployed as soon as possible until the coverage rate of drone sites reaches about 70% of the overall scenario so as to maximize the utility of drone site deployment.

**Fig 7:**
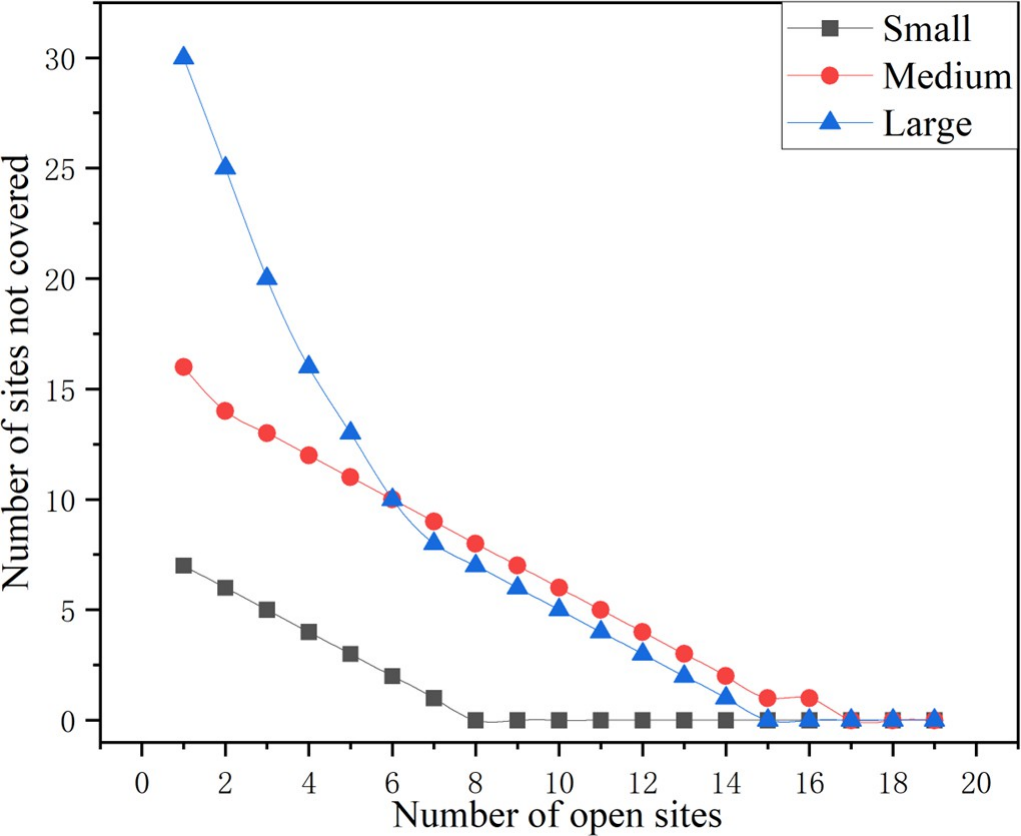
Map of the number of uncovered sites at progressively open sites at various scales.

The rate of decline in the number of uncovered sites is fastest for all sizes during the initial phase (0–5 open sites). After the initial phase, the rate of decline slows down, suggesting that adding more sites has diminishing returns in terms of reducing the number of uncovered sites. As shown in Fig 7, increasing the number of open sites significantly reduces the number of initial uncovered sites, but this gain diminishes as the number of sites increases further. The large scale starts with the highest number of uncovered sites and has the most significant initial decline, but even after 20 sites are open, it is still higher than the number of uncovered sites for the other two scales, but the difference is already very small.

**Fig 8:**
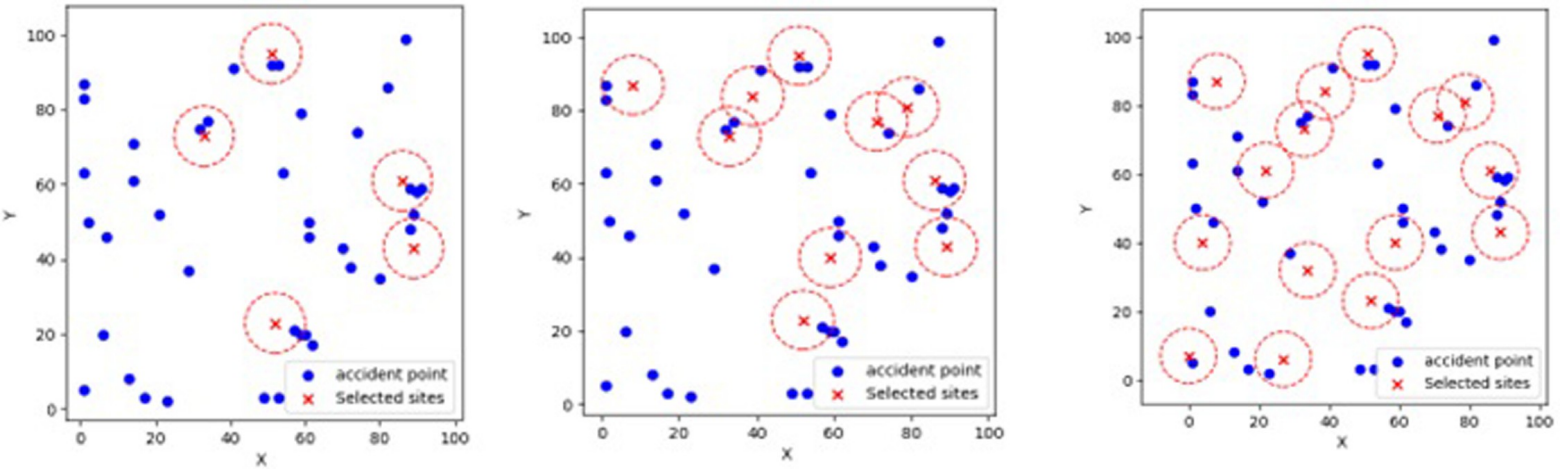
UAV site coverage in large-scale scenarios. (a) Number of Sites = 5. (b) Number of Sites = 10. (c) Number of Sites = 15.

Therefore, in our subsequent analyses, we chose the number of open sites to be 15. This number of open sites covers as many accident points as possible while controlling costs. To demonstrate the algorithm’s effects on different numbers of sites and spatial layouts more intuitively, we selected three cases of site numbers (5, 10, 15), solved them in a large-scale scenario, and presented the visualization results as shown in Fig 8:

### 5.4 Parameter sensitivity analysis

Sensitivity analysis lies in assessing the impact of different parameters on model output and performance. By gradually adjusting key parameters, such as coverage radius, accident probability distribution, etc., sensitivity analysis helps us to understand the model’s response to changes in these parameters, thus revealing the stability and robustness of the model. In practice, sensitivity analysis can guide decision-makers in choosing the optimal parameter settings and ensure that the model maintains good performance under different scenarios. Firstly, the coverage radius is analyzed, and we increase the coverage radius from 2 to 20 by 0.5 units each time and record the optimal solution quality and computation time under each coverage radius. Using the large-scale calculus, the number of open sites is 15, and the number of incident points is 40, and the solution results are shown in Table 9.

**Table 9:** Coverage radius sensitivity analysis table.

coverage radius	optimal solution quality	computation time(s)	coverage radius	optimal solution quality	computation time(s)
2	158.2	7.5	11.5	88.3	13.1
2.5	150.6	7.8	12	87.6	13.4
3	143.3	8	12.5	87	13.7
3.5	136.7	8.3	13	86.5	14
4	130.1	8.6	13.5	86.1	14.3
4.5	124.5	8.9	14	85.8	14.6
5	119	9.2	14.5	85.6	14.9
5.5	114.3	9.5	15	85.5	15.2
6	110.1	9.8	15.5	85.4	15.5
6.5	106.3	10.1	16	85.4	15.8
7	103	10.4	16.5	85.5	16.1
7.5	100.2	10.7	17	85.6	16.4
8	97.8	11	17.5	85.8	16.7
8.5	95.8	11.3	18	86.1	17
9	94	11.6	18.5	86.4	17.3
9.5	92.5	11.9	19	86.8	17.6
10	91.2	12.2	19.5	87.3	17.9
10.5	90.1	12.5	20	87.8	18.2
11	89.1	12.8			

**Fig 9:**
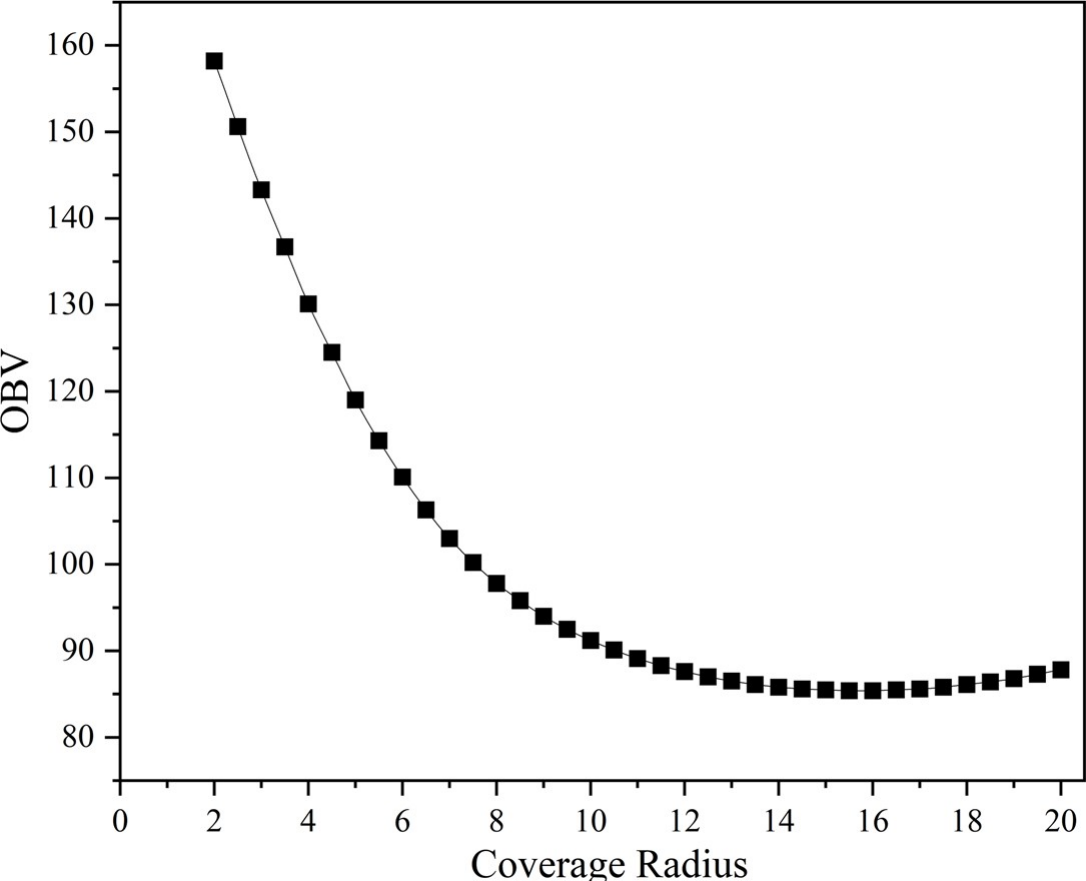
Coverage radius sensitivity analysis.

The optimal solution quality increases significantly with the coverage radius, gradually decreasing from 158.2 at a coverage radius of 2 to 90.1 at a coverage radius of 10.5, as shown in Table 9. After the coverage radius reaches 12, the optimal solution quality tends to stabilize, fluctuating between 85 and 88 with little change. As the coverage radius increases, the computation time increases gradually, from 7.5 seconds at a coverage radius of 2 to 18.2 seconds at a coverage radius of 20. The computation time shows a linear growth trend, increasing by approximately 0.3 seconds for every 0.5 increase in coverage radius. The optimal solution quality and computation time reach an equilibrium point at a coverage radius of about 8 when the optimal solution quality is 97.8 and the computation time is 11 seconds. As shown in Fig 9, the coverage radius can be chosen between 8 and 10 in order to find an efficient balance between optimal solution quality and computation time. In this range, the optimal solution quality is more stable, and the computation time remains within an acceptable range. According to the analysis, in order to maintain high optimal solution quality while controlling the computation time, it is recommended to choose a coverage radius between 8 and 10 for optimization. In practical applications, the specific choice of coverage radius still needs to be weighed according to the actual demand and computational resources and should be converted using the actual scenario scale compared with the arithmetic unit, but in some larger scenarios, it may be affected by issues such as the range of different types of UAVs. The management insights gained are that the cost of drone services decreases significantly with the drone coverage radius, but the utility slows down as the drone coverage radius expands further, so more advanced drone facilities should be actively pursued, but the need is not extreme.

**Fig 10:**
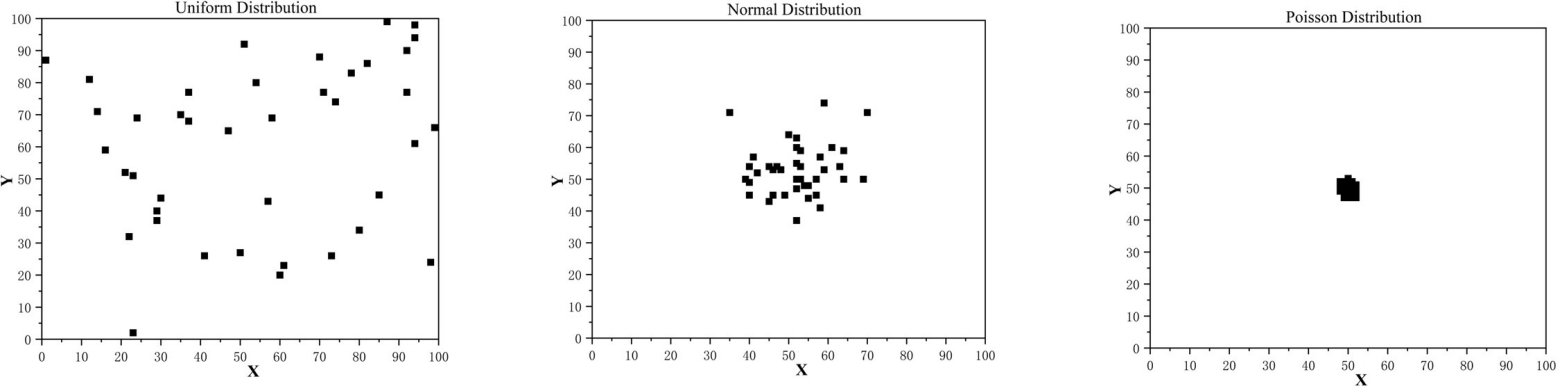
Sensitivity analysis of accident point distribution. (a) Uniform Distribution. (b) Normal Distribution. (c) Poisson Distribution.

When we performed the sensitivity analysis of accident point distribution, we used uniform, normal, and Poisson distributions to generate accident points and observe the performance of the model under different distribution conditions. As shown in Fig 10, with uniform distribution, the accident points are uniformly distributed in the region, which usually results in a shorter computation time for the optimization model and a higher quality of the optimal solution. The normal distribution, on the other hand, is concentrated in a certain region, resulting in the model requiring more adjustments to cover the accident points in the concentrated region, and the computation time and complexity increase. With the Poisson distribution, the accident points show more concentrated characteristics, further increasing the difficulty of coverage and computation time.

As shown in Table 10, from the viewpoint of the calculation time of the three distributions, the more dispersed the distribution, the less calculation time, and the smaller the calculation burden. In terms of solution quality, the more dispersed the distribution, the higher the solution quality, i.e., UAVs are more likely to deal with more dispersed traffic accidents and are not suitable for use in densely populated traffic accidents.

**Table 10:** Sensitivity analysis of accident point distribution.

Distribution situation of accident probabilities	Calculating time (s	Optimal solution quality
Uniform distribution	9.2	119
Normal distribution	10.5	125.4
Poisson distribution	11.1	130.2

## 6 Conclusion

This paper primarily examines the application of drones in traffic accident assessment and processing and presents an optimization issue regarding the strategic pre-positioning of drone stations. The primary challenge of this problem stems from the uncertainty of the spatial distribution of future traffic accidents. To address this challenge, we have proposed a drone pre-site selection model after road discretization. Based on our experimental outcomes, the following insights have been derived. Firstly, within a certain range, when the demand scope and quantity at accident points are in the increasing phase, it does not impact the efficacy of drone station deployment. Thus, in the actual deployment process, efforts should be made to deploy as soon as possible until the coverage rate of drone stations reaches approximately 70% of the overall scenario, maximizing the deployment efficacy of drone stations. Secondly, the service cost of drones will decline significantly with the increase of the drone coverage radius, but the efficacy will slow down with further expansion of the drone coverage radius. Therefore, more advanced drone facilities should be actively pursued, although this demand is not extreme. Thirdly, drones are more adept at handling dispersed traffic accidents rather than concentrated ones.

In terms of the potential future research directions of drone applications in traffic accidents, the specialization and clustering of drone types have raised new expectations for further enhancements in traffic accident handling. Additionally, with the implementation of intelligent roads, it is worthy of in-depth exploration on how to combine the drone system with intelligent road applications to better serve the transportation network. Our future research endeavors will focus on improvements in these aspects.

## Supporting information

S1 File (XLSX)
